# Sphingosine-1-Phosphate/Sphingosine-1-Phosphate Receptor 2 Axis Can Promote Mouse and Human Primary Mast Cell Angiogenic Potential through Upregulation of Vascular Endothelial Growth Factor-A and Matrix Metalloproteinase-2

**DOI:** 10.1155/2016/1503206

**Published:** 2016-01-14

**Authors:** Alena Chumanevich, Piper Wedman, Carole A. Oskeritzian

**Affiliations:** Department of Pathology, Microbiology and Immunology, University of South Carolina School of Medicine, Building 2, Room C10, 6439 Garners Ferry Road, Columbia, SC 29209, USA

## Abstract

Mast cells (MC) are present in most vascularized tissues around the vasculature likely exerting immunomodulatory functions. Endowed with diverse mediators, resident MC represent first-line fine-tuners of local microenvironment. Sphingosine-1-phosphate (S1P) functions as a pluripotent signaling sphingolipid metabolite in health and disease. S1P formation occurs at low levels in resting MC and is upregulated upon activation. Its export can result in type 2 S1P receptor- (S1PR2-) mediated stimulation of MC, further fueling inflammation. However, the role of S1PR2 ligation in proangiogenic vascular endothelial growth factor- (VEGF-) A and matrix metalloproteinase- (MMP-) 2 release from MC is unknown. Using a preclinical MC-dependent model of acute allergic responses and* in vitro* stimulated primary mouse bone marrow-derived MC (BMMC) or human primary skin MC, we report that S1P signaling resulted in substantial amount of VEGF-A release. Similar experiments using* S1pr2*-deficient mice or BMMC or selective S1P receptor agonists or antagonists demonstrated that S1P/S1PR2 ligation on MC is important for VEGF-A secretion. Further, we show that S1P stimulation triggered transcriptional upregulation of VEGF-A and MMP-2 mRNA in human but not in mouse MC. S1P exposure also triggered MMP-2 secretion from human MC. These studies identify a novel proangiogenic axis encompassing MC/S1P/S1PR2 likely relevant to inflammation.

## 1. Introduction

Mast cells (MC) convey immunomodulatory functions through their unique ability to release, after activation, many mediators including cytokines, chemokines, and enzymes, some of which are prestored in cytoplasmic granules at homeostasis [1]. MC are* bona fide* sentinels present in most vascularized tissues prior to trauma, equipped with a vast selection of surface receptors, therefore sensing and quickly responding to local alterations [2]. Notorious for their key contributions to allergic responses, MC influence the course and chronicity of many inflammatory disorders [3–5].

We discovered that immunoglobulin E- (IgE-) dependent MC activation releases sphingosine-1-phosphate (S1P), a bioactive sphingolipid mediator produced by sphingosine kinases that serves to further propagate MC-mediated inflammatory response [3, 4, 6]. S1P exerts its pleiotropic actions imparted by ligation to five G-protein-coupled receptors (GPCRs), S1PR1–S1PR5, with subtype-specific distinct repertoire of heterotrimeric G protein coupling, in combination with tissue- and cell-type-specific receptor expression patterns [7]. Our recent studies established MC as critical to the onset of acute pulmonary allergic response [3] through autocrine/paracrine binding of S1P on MC surface S1P receptor type 2 (S1PR2). This interaction led to MC-derived T-cell-attracting chemokine release partly through signal transducer and activator of transcription 3 (Stat3) signaling [4]. Suppression of MC S1PR2 signaling by S1PR2 genetic ablation or pharmacological antagonism significantly impaired T-cell recruitment through decreased release of T-cell chemoattractants in activated MC supernatants. Moreover, eliminating extracellular S1P with a monoclonal antibody (mAb) that binds and neutralizes S1P mitigated* ex vivo* and* in vivo* allergic MC activation, yielding significant inhibition of inflammatory infiltration and chemokine detection [4]. Of note, this mAb was shown to be effectively antiangiogenic in mouse xenograft and allograft tumor models [8, 9] as well as in a mouse model of wet age-related macular degeneration, that is, choroidal neovascularization [10].

Angiogenesis, or the formation and maintenance of blood vessel structures, is essential for the physiological functions of tissues and for the progression of diseases such as cancer and inflammation [11, 12]. S1P stimulates endothelial cell proliferation and survival, migration, and capillary-like tube formation via S1PR1 and S1PR3* in vitro*, which is indicative of its angiogenic activity [13, 14]. S1P also maintains endothelial barrier function via S1PR1 [15, 16].

In addition to histamine, a potent vasoactive amine triggering vascular leakage and edema, and S1P, MC can generate an array of angiogenic factors, including vascular endothelial growth factor-A (VEGF-A), upon cross-linking of surface bound IgE on their high-affinity receptors (Fc*ε*RI) with allergen (Ag) and other stimuli [17–20]. VEGF-A regulates angiogenesis and vascular permeability by activating 2 receptors, VEGFR-1 (Flt-1) and VEGFR-2 (KDR/Flk1 in mice) [21]. Interestingly, S1P can activate VEGFR-2 in the absence of its conventional ligand VEGF by receptor cross talk [22]. Ligation of S1PR2 on the cell membrane can induce ERK1/2 activation through activation of Gi protein. Activation of Gi can lead to Src activation that results in the phosphorylation of VEGFR-2. This transactivation of VEGFR-2 also contributes to vascular remodeling [23].

In the present study, we further investigated the role of S1P signaling through S1PR2 on MC and show for the first time that S1P stimulation of primary mouse and human MC results in the release of substantial amounts of VEGF-A. The relevance of these findings was substantiated using genetically S1PR2-deficient and congenic wild-type mouse model of acute allergic response [3, 4], as well as selective pharmacological receptor agonist and antagonist [3, 4, 24]. Moreover, our results provide new molecular insights pertaining to S1P-regulated VEGF-A release from primary human MC. Finally we report that S1P-dependent MC activation induces the release of matrix metalloproteinase- (MMP-) 2, endowed with extracellular and connective tissue matrix degrading properties [25], therefore potentially also contributing to neovascularization in inflammatory and cancer settings.

## 2. Materials and Methods

### 2.1. Mice

Age- and gender-matched C57BL6/J mice were obtained from the National Institutes of Health (NIH) National Cancer Institute (Frederick, MD, USA). S1PR2 knockout mice (Taconic Biosciences, Inc., Hudson, NY) and corresponding wild-type (WT) mice were on a mixed 129/SvEV-C57BL/6 background. All mice were maintained in a pathogen-free facility. Studies were performed in accordance with institutional animal care and use committee guidelines.

### 2.2. Reagents

Dinitrophenyl- (DNP-) specific mouse IgE was a generous gift from Dr. Daniel Conrad (VCU, Richmond, VA). DNP-human serum albumin (HSA) and ionomycin were obtained from Sigma-Aldrich (St Louis, MO). S1P was purchase from Enzo Life Sciences (Farmingdale, NY). JTE-013 and CYM-5442 were purchased from Tocris/Bio-Techne (Minneapolis, MN).

### 2.3. Human Skin and Mouse Bone Marrow-Derived Mast Cells

Human skin MC and mouse bone marrow-derived mast cells (BMMC) were isolated and cultured essentially as previously described [26, 27] and were more than 98% pure. Human MC and mouse BMMC were sensitized overnight with 1 *μ*g/mL DNP-specific mouse IgE, washed to remove excess unbound IgE, and stimulated with 30 or 20 ng/mL DNP-HSA (Ag), respectively, for 24 hours. Ionomycin (1 *μ*M), a receptor-independent stimulus, and S1P (100 nM) were also applied for 24 hours. Of note, vehicle consisted of DMSO/PBS-4 mg/mL fatty acid-free bovine serum albumin. All supernatants were collected after 24 hours of stimulation. Each experiment was performed at least three times, with triplicate determinations. For mRNA analysis, 3–5 × 10^6^ human skin MC from 5 different donors were incubated for the indicated times in the presence or absence (vehicle, or culture medium alone) of S1P (100 nM).

### 2.4. Acute Mast Cell-Dependent Allergic Response

All injections were performed intraperitoneally in a final volume of 100 *μ*L, as previously described [3, 4]. Briefly, mice were injected with DNP-specific IgE and 12 hours later with Ag in PBS. Mice were euthanized 2 hours later and blood was immediately collected by cardiac puncture for serum analysis.

### 2.5. VEGF-A and MMP-2 Measurements

Human and mouse angiogenic factors were measured by ELISA, according to manufacturer's instructions (Bio-Techne, Minneapolis, MN).

### 2.6. Quantitative PCR Analysis

Total RNA was isolated and purified with the miRNeasy Kit (Qiagen, Valencia, CA), following the manufacturer's procedure. The iScript cDNA synthesis kit (Bio-Rad, Hercules, CA) was used according to the manufacturer's specifications to reverse-transcribe cDNA. QPCR was performed on a CFX Connect (Bio-Rad) with SensiFAST SYBR No-ROX Kit (Bioline). The following primers were used for real-time PCR amplification: human VEGF-A, forward primer 5′-AGGCCAGCACATAGGAGA-3′ and reverse primer 5′-ACCGCCTCGGCTTGTCACAT-3′; human MMP-2, forward primer 5′-TACAGGATCATTGGCTACACACC-3′ and reverse primer 5′-GGTCACATCGCTCCAGACT-3′; human GAPDH, forward primer 5′-TTGAGGTCAATGAAGGGGTC-3′ and reverse primer 5′-GAAGGTGAAGGTCGGAGTCA-3′; mouse VEGF-A, forward primer 5′-GGCCTCCGAAACCATGAACT-3′ and reverse primer 5′-CTGGGACCACTTGGCATGG-3′; mouse MMP-2, forward primer 5′-ACCTGAACACTTTCTATGGCTG-3′ and reverse primer 5′-CTTCCGCATGGTCTCGATG-3′; and mouse GAPDH, forward primer 5′-CAGAAGGGGGCGGAGATGA-3′ and reverse primer 5′-AGGCCGGTGCTGCTGAGTATGTC-3′. The real-time PCR conditions were as follows: initial step at 95°C for 10 min and cycles (*n* = 40) consisted of 10 s at 95°C, followed by 20 s annealing/extension at 59°C and extension at 72°C. All reactions were performed in duplicate. Data were analyzed with CFX Manager software (Bio-Rad) and are normalized expression directly proportional to the amount of mRNA of the target genes VEGF-A and MMP-2 relative to the amount of mRNA of the reference gene, GAPDH. Primers were synthesized and purchased from Thermo Fisher Scientific, Inc. (Waltham, MA), with melting temperatures ranging from 59.9 to 64.5°C.

### 2.7. Statistical Analysis

Data are expressed as means ± SEM and were analyzed by using unpaired two-tailed Student's *t*-test for comparison of two groups (Prism 6; GraphPad Software, La Jolla, CA). Significance for statistical tests is shown in figures. Experiments were repeated at least three times in duplicate or triplicate with consistent results.* In vivo* experiments were repeated twice (*n* = 5-6 mice per experimental group).

## 3. Results

### 3.1. MC- and IgE-Dependent Acute Allergic Reactions Trigger Systemic VEGF-A Detection in WT Mice That Is Significantly Mitigated in* S1pr2*-Null Mice

**Figure 1:**
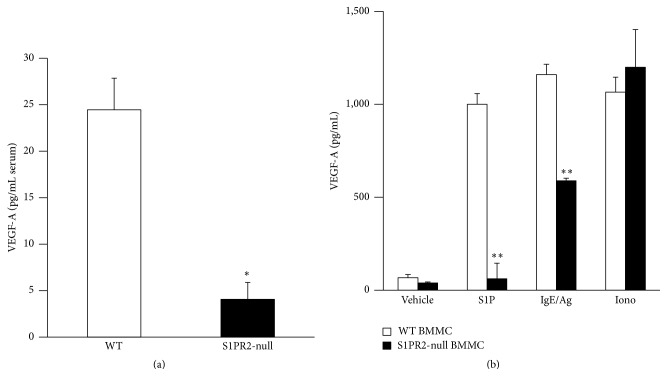
Role of S1P/S1PR2 signaling in VEGF-A secretion. (a) Blood was collected from allergenically challenged WT (open bar, *n* = 5-6 mice) and S1PR2-null (black bar, *n* = 5-6 mice) mice, euthanized 2 hours after Ag challenge, and serum VEGF-A levels were measured in duplicate determination for each animal. (b) Bone marrow-derived mast cells (BMMC, three independent populations) from both genotypes were stimulated for 24 hours with vehicle (DMSO/PBS/4 mg/mL fatty acid-free BSA), S1P (100 nM), IgE/Ag, or ionomycin (Iono) and VEGF-A was measured in MC supernatants, in duplicate determinations. Error bars show standard error of means. ^*∗*^ *p* < 0.05, ^*∗∗*^ *p* < 0.005.

We previously reported that substantial levels of circulating chemokines were detected in the serum of WT mice 2 hours after eliciting a MC-dependent allergenic challenge [4]. We also showed that chemokine production was significantly decreased in the absence of extracellular S1P [4] or in mice genetically ablated for* S1pr2* or pretreated with a selective S1PR2 antagonist [3]. Similarly, circulating levels of VEGF-A were measured in the serum of WT and* S1pr2*-null mice in the same preclinical model.  Figure 1(a) shows that as early as 2 hours after Ag administration significant levels of VEGF-A were detected in the serum of sensitized WT while the absence of* S1pr2* severely mitigated serum VEGF-A levels in sensitized* S1pr2*-deficient mice. Of note, VEGF-A was undetectable in the serum of either genotype prior to allergenic challenge (data not shown). We further substantiated the relevance of* S1pr2* in VEGF-A secretion by stimulating* in vitro* mouse BMMC from both genotypes and measuring VEGF-A in activated BMMC supernatants.  Figure 1(b) shows that S1P (100 nM) and IgE/Ag stimulation trigger significant release of VEGF-A in the supernatants of activated WT BMMC collected after 24 hours that was significantly decreased in* S1pr2*-deficient BMMC, further emphasizing the importance of S1PR2 signaling on MC for VEGF-A secretion. Of note, ionomycin, a calcium ionophore and receptor-independent MC stimulus, also triggered significant VEGF-A release from BMMC that was unaffected by the absence of S1PR2.

### 3.2. *In Vitro* S1P- or IgE/Ag-Mediated Stimulation of Human Primary Mast Cells Results in the Release of Angiogenic Factors VEGF-A and MMP-2

**Figure 2:**
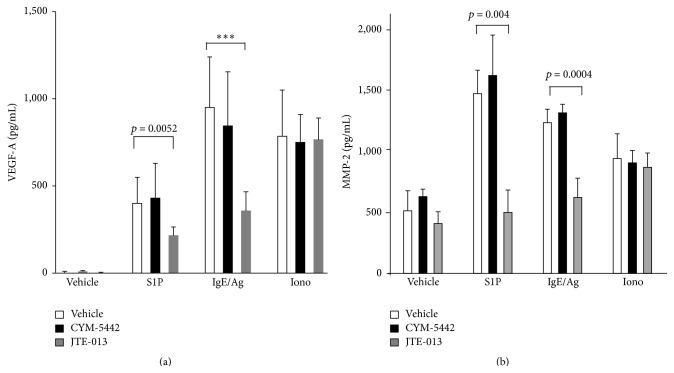
Human MC secrete proangiogenic factors upon exposure to S1P in a S1PR2-dependent manner. (a) VEGF-A levels were measured in the supernatants of activated human MC in the absence (open bars, vehicle (DMSO/PBS/4 mg/mL fatty acid-free BSA)) or presence of a 30-minute pretreatment with CYM-5442 (filled bars, S1PR1 agonist, 1 *μ*M) or with JTE-013 (gray bars, S1PR2 antagonist, 1 *μ*M), followed by the indicated stimuli similar to Figure 1(b). (b) Matrix metalloproteinase- (MMP-) 2 levels were measured in supernatants of MC activated exactly as described in Figure 1(b). All supernatants were collected 24 hours after stimulation. Activation experiments were conducted using five independent human skin MC populations generated from five donors. Activation was conducted in triplicate determinations and measurements were conducted in duplicate determinations for each individual determination. When reaching significance, statistics are indicated in each figure. Error bars show standard error of means. ^*∗∗∗*^ *p* < 0.0001.

Next, to validate the physiological relevance of these findings, primary human mast cells were assessed for their ability to release angiogenic factors. As shown in Figure 2(a), addition of exogenous S1P or Ag to sensitized human skin MC stimulates VEGF-A secretion that occurs at very low levels spontaneously. Ionomycin, a calcium ionophore and receptor-independent MC stimulus, also triggers VEGF-A release from human primary skin MC. The pretreatment of cells for 30 minutes in the presence of CYM-5442, a selective S1PR1 agonist, did not induce any VEGF-A secretion from MC on its own. Furthermore, exposure to CYM-5442 (1 *μ*M) prior to S1P or IgE/Ag did not alter the levels of VEGF-A released by either of these two stimuli. However, pretreating MC with JTE-013, a potent and selective antagonist for S1PR2, significantly inhibited both S1P- and IgE/Ag-mediated VEGF-A release from MC.  Figure 2(b) demonstrates that S1P, IgE/Ag, and ionomycin stimuli also induce the secretion of large amounts of MMP-2 from human skin MC. Similar to Figure 2(a), pretreatment with CYM-5442 prior to stimulant exposure did not prevent MMP-2 secretion from human MC (Figure 2(b)). By contrast, preexposure to JTE-013, a pharmacological antagonist for S1PR2, significantly mitigated S1P- and IgE/Ag-dependent release of MMP-2 from human MC (Figure 2(b)), establishing that MC-derived VEGF-A and MMP-2 secretion requires functional MC-expressed S1PR2. It is noteworthy that S1P, itself potentially proangiogenic, promoted the release of VEGF-A and MMP-2 from human skin MC.

### 3.3. S1P Stimulation Transcriptionally Upregulates VEGF-A and MMP-2 mRNA Expression in Human but Not in Mouse Primary Mast Cells

**Figure 3:**
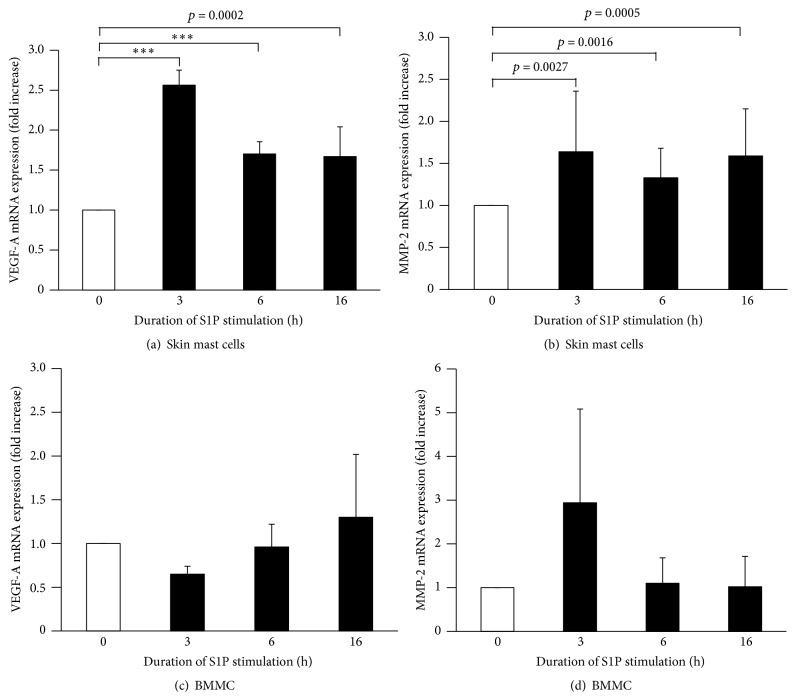
Effect of S1P on VEGF-A and MMP-2 mRNA expression. Human (a, b) and mouse (c, d, BMMC) MC were stimulated in the presence of S1P (100 nM) for different periods of time at which VEGF-A (a, c) and MMP-2 (b, d) mRNA levels were measured by QPCR. mRNA was prepared from 3 independent BMMC and 5 independent human MC populations, in duplicate. Kinetics of S1P stimulation was repeated 3 to 4 times; each QPCR experiment was performed in duplicate determination for each individual sample. When reaching significance, statistics are indicated in each figure. Error bars show standard error of means. ^*∗∗∗*^ *p* < 0.0001.

Since* in vitro* stimulation of primary MC is conducted for 24 hours, we next sought to determine whether S1P could also activate VEGF-A and MMP-2 mRNA production during this time. To this end, primary human skin MC were stimulated for different periods of time in the presence of S1P (100 nM) and kinetics of VEGF-A (Figure 3(a)) and MMP-2 (Figure 3(b)) mRNA expression was investigated. Figures 3(a) and 3(b) show that stimulation of human skin MC for 3 hours triggered a significant increase in both VEGF-A and MMP-2 mRNA expression that decreased with increased incubation time but remained higher than in the absence of stimulation. Results were consistent for human skin MC derived from 5 different donors. In contrast, similar stimulation of BMMC did not result in upregulation of transcription for either of these two genes (Figures 3(c) and 3(d)). These results were replicated using three different BMMC populations.

## 4. Discussion

Inflammation first manifests itself with vascular alterations, including increased vascular permeability and edema [28]. MC are tissue-dwelling cells located around blood vessels and can release a number of vasoactive mediators that act directly on the vasculature, promoting vasodilation, increased endothelial permeability, and subsequent extravascular leakage of proteins and fluid [5]. Among the panoply of MC-derived bioactive mediators affecting the endothelium, histamine is released within minutes after activation [28]. We and others have shown that, upon allergenic activation of MC, concomitant sphingosine kinase activation occurs, leading to phosphorylation of sphingosine to rapidly produce S1P [6, 29–31]. We have also reported that S1P could be exported out of and act on the MC that produced it in an autocrine/paracrine manner [32] by ligation to S1PR1 or S1PR2, the two S1PR subtypes expressed on the MC surface [31]. This creates an inflammatory amplification loop that we have shown is essential to the onset of acute allergic inflammation [3, 4, 31, 33]. Importantly, S1P is also constitutively released from human MC in the absence of stimulus [32]. This is an important point, as MC could be a source of local S1P at homeostasis in tissues, further prominent in inflamed sites [33].

Angiogenesis is an important feature of development and perpetuation of allergic inflammation [34]. The levels of VEGF-A are increased in the airways of asthmatic patients and MC constitute the majority of VEGF-A positive cells in bronchial biopsies from asthmatics [35]. We report for the first time that S1P stimulation of mouse and human primary MC is a potent inducer of VEGF-A release. Many studies have reported MC as a major source of VEGF [35–39]. We had previously shown that* S1pr2* deficiency in mice or in MC drastically reduced acute allergic responses and early T-cell recruitment [3, 4]. Remarkably, our data indicate that acute allergen challenge of sensitized mice triggered immunodetectable levels of circulating VEGF-A as early as two hours after Ag challenge. In comparison, sensitized* S1pr2*-null mice display impaired systemic VEGF-A at the same time point after Ag challenge, suggesting an important function of S1PR2 signaling in VEGF-A secretion. Interestingly, similar findings have been reported in neuroblastoma cells, which also exhibited enhanced formation of S1P that ligated S1PR2 to induce VEGF expression [40]. In agreement with our study, the effect of S1P on VEGF mRNA expression also occurred at the transcriptional level [40] in human but not in mouse MC, suggesting contrasted and species-specific mechanisms regulating transcription. Supporting our findings, S1PR2 has been shown to be essential for normal and pathological angiogenesis [41, 42]. Our results are important because they show for the first time that MC/S1P/S1PR2 axis could promote proangiogenic VEGF-A secretion from MC.

The cross talk between S1P and VEGF signaling has been identified more than a decade ago but was typically assigned to S1PR1 [22, 43]. Whether they directly affected each other remained elusive. Our current study establishes that S1P can indeed stimulate VEGF-A secretion from primary MC through ligation to S1PR2, as CYM-5442, a selective pharmacological agonist of S1PR1 [24], did not induce VEGF-A secretion nor did it alter S1P- or IgE-Ag-mediated VEGF-A release. By contrast, JTE-013, a potent and selective antagonist for S1PR2, significantly impaired S1P and IgE/Ag-mediated VEGF-A secretion from primary MC. Of note and although S1PR2 functions in cancer are still obscure [40, 44, 45], our data suggest that S1P/S1PR2 signaling in MC may promote local VEGF production and therefore angiogenesis, potentially linking inflammation to cancer.

Moreover, S1P and IgE/Ag we show can stimulate the secretion of MMP-2 from human primary MC possibly extending their contribution as connectors of inflammation to metastatic cancer [46, 47]. Interestingly, active forms of MMP have been associated with the development of vasogenic edema and disruption of blood vessel integrity in stroke reperfusion therapy and S1PR2 has recently been shown to be critical in MMP-9 activation [48]. MC have long been known to produce MMP-9 [33, 49, 50] and MMP-2 [33, 51]. In agreement, we found that S1PR2 ligation by S1P resulted in MMP activation in primary MC.

It is important to mention that MC harbor nonconventional yet proangiogenic granule-associated mediators, including tryptase and chymase [46], particularly because they can activate latent forms of MMP [52]. It is tempting to speculate that S1P can directly activate MC to release MMP-2. Alternatively, because (1) we and others have previously reported that S1P contributes to MC activation and degranulation and (2) tryptase and chymase are granule-associated and as such released from MC upon activation, we can hypothesize an indirect influence of S1P on active MMP-2 release from MC through tryptase and/or chymase exocytosis, which, in turn, could activate MMP-2. These equally intriguing hypotheses are not mutually exclusive and highlight potentially new mechanistic insights warranting further investigations relevant to inflammation and cancer.

In sum, our findings demonstrate that S1P produced by MC in inflammatory settings can induce VEGF-A release from MC by ligation to S1PR2, further emphasizing a critical role of MC as pivotal cells influencing the conversion of acute inflammation to chronicity and perhaps carcinogenesis. Moreover, based on our previous reports demonstrating a downregulatory effect of* S1pr2* deficiency or functional antagonism on MC responsiveness, this report further suggests the importance of fine-tuning S1P signaling through MC S1PR2 as a relevant anti-inflammatory strategy and, potentially, a novel approach to chemoprevention of inflammation.
